# Receptor Structure Shapes Host Range but Incompletely Predicts High Activity in *Klebsiella pneumoniae* Phage Cocktails

**DOI:** 10.3390/antibiotics15090900

**Published:** 2026-09-12

**Authors:** Roman B. Gorodnichev, Anastasiia O. Krivulia, Maryam O. Sidorskaia, Maria A. Kornienko, Narina K. Abdraimova, Maja V. Malakhova, Marina V. Zaychikova, Dmitry A. Bespiatykh, Egor A. Shitikov

**Affiliations:** 1 Department of Biomedicine and Genomics, Lopukhin Federal Research and Clinical Center of Physical-Chemical Medicine of Federal Medical Biological Agency, Moscow 119435, Russia; kimnimka@gmail.com (A.O.K.); sidorskaja.maryam@yandex.ru (M.O.S.); kornienkomariya@gmail.com (M.A.K.); abdraimovanarina@gmail.com (N.K.A.); maja_m@mail.ru (M.V.M.); marinaz15@yandex.ru (M.V.Z.); d.bespiatykh@gmail.com (D.A.B.); eshitikov@mail.ru (E.A.S.); 2 Moscow Center for Advanced Studies, Moscow 123592, Russia

**Keywords:** *Klebsiella pneumoniae*, bacteriophage therapy, phage cocktails, phage susceptibility testing, host range, post-adsorption resistance, rational cocktail design

## Abstract

Background. Rational design of phage cocktails typically relies on the lytic spectrum. However, susceptibility criteria vary widely among studies, ranging from qualitative lysis in spot tests to quantitative endpoints based on serial-dilution titration or efficiency of plating. Methods. We investigated this discrepancy using four commercial phage cocktails and two capsule-specific monophages against a clinically representative collection of 448 *Klebsiella pneumoniae* isolates comprising 56 capsule types, collected in 2018–2025 from 12 medical centers. Using a modified Gratia titration assay, we defined host range (HR) as specific lysis at any dilution and putative therapeutic applicability (PTA) as lysis at dilutions corresponding to ≥10^6^ plaque-forming units per mL. Results. We identified a systematic discrepancy between these two measures of cocktail efficacy. HR coverage reached 64%, whereas PTA was significantly lower, with a median HR–PTA difference of 36%. This discrepancy persisted for capsule-specific monophages tested against KL2 isolates (*n* = 69), indicating that neither nominal capsule matching nor low component titres fully explained the loss of activity. This pattern provides indirect functional evidence that post-adsorption barriers contribute to the HR–PTA discrepancy, although adsorption and intracellular antiphage mechanisms were not assessed directly. Capsule type was the principal and most robust predictor of efficacy, whereas spatiotemporal factors, including year and medical centre, had only a weak effect on PTA. Conclusions. Capsule matching can therefore help estimate population-level coverage but does not fully predict high-titre activity. Phage selection should incorporate serial-dilution testing rather than rely on spot-test lysis alone. Cocktail design should account for both receptor coverage and functional activity against representative isolates within individual capsule types.

## 1. Introduction

Antimicrobial resistance is considered one of the major threats to global health and is projected to be associated with up to 10 million deaths annually by 2050 [1]. This threat has renewed interest in phage therapy, an approach developed in the early twentieth century that exploits the ability of bacteriophages to infect and lyse bacterial cells.

Two principal approaches to implementing phage therapy have emerged: the use of standardized, pre-formulated phage cocktails and individualized treatment with phages selected for activity against the patient’s bacterial isolate. Pre-formulated, commercial cocktails are ready-to-use preparations standardized in accordance with current pharmacopoeial requirements [2]. However, their clinical efficacy has not been convincingly demonstrated in rigorous blinded, placebo-controlled trials [3,4]. Personalized phage therapy can achieve high lytic activity and has been associated with favorable clinical outcomes in numerous case reports [5], but it is time- and resource-intensive, logistically demanding, while its individualized nature limits the degree of standardization achievable for conventional pharmaceutical products [6].

Against this background, rational cocktail design has emerged to bridge the practicality of predefined formulations and the targeted activity of personalized phage therapy [7]. This approach typically selects phages according to their lytic spectra, with the aim of covering the greatest diversity of target bacteria within a hospital or region using the smallest possible set of active bacteriophages. Nevertheless, standards for assessing lytic spectra and the criteria used to select bacteriophages for inclusion in cocktails vary among studies [7,8,9].

Variation among protocols for assessing phage activity complicates the interpretation and comparison of results [10,11,12,13]. In practice, bacterial susceptibility is often assessed solely by recording lysis, for example in a spot test [10]. However, lysis alone does not guarantee therapeutically relevant activity, which generally requires productive phage replication despite post-adsorption barriers [14]. Although several pioneering studies have considered the effects of these barriers [15,16,17], their contribution to the loss of phage activity has not been distinguished from that of insufficient receptor coverage. This uncertainty limits the extent to which findings obtained using a local strain panel can be extrapolated to other clinical collections.

*Klebsiella pneumoniae* provides a useful model for evaluating how site-specific adsorption and post-adsorption barriers affect bacteriophage efficacy. In many *K. pneumoniae* phages, adsorption is mediated by capsular polysaccharides targeted by phage depolymerases [18,19]. Capsular polysaccharides are well-characterized epidemiological markers, enabling bacterial isolates to be stratified when assessing phage activity. The capsule is also a major determinant of *K. pneumoniae* virulence because it protects bacterial cells from immune responses and phagocytosis [20].

The high clinical importance of *K. pneumoniae* further supports the use of this model. Carbapenem-resistant strains are included in the World Health Organization list of priority bacterial pathogens [21]. Infections caused by *K. pneumoniae* are associated with approximately 800,000 deaths annually, of which up to 80% are attributable to antibiotic-resistant strains [21]. The widespread circulation of these strains compromises the effectiveness of conventional antimicrobial therapy and reinforces the need for alternative treatment strategies [22].

Here, we aimed to quantify the discrepancy between the nominal host range, defined by the presence of specific lysis, and lysis retained at a predefined *in vitro* titre threshold in a clinically representative collection of *K. pneumoniae* isolates stratified by capsule type. We compared broad-spectrum commercial phage cocktails with capsule-specific monophages to assess whether capsule matching alone could predict high-titre lysis. We also assessed the extent to which activity profiles were associated with spatiotemporal factors, including year of isolation and medical centre, to determine the limits of extrapolating these findings to other clinical populations.

## 2. Results

### 2.1. Capsule-Type Distribution in the Klebsiella pneumoniae Isolate Collection

**Figure 1:**
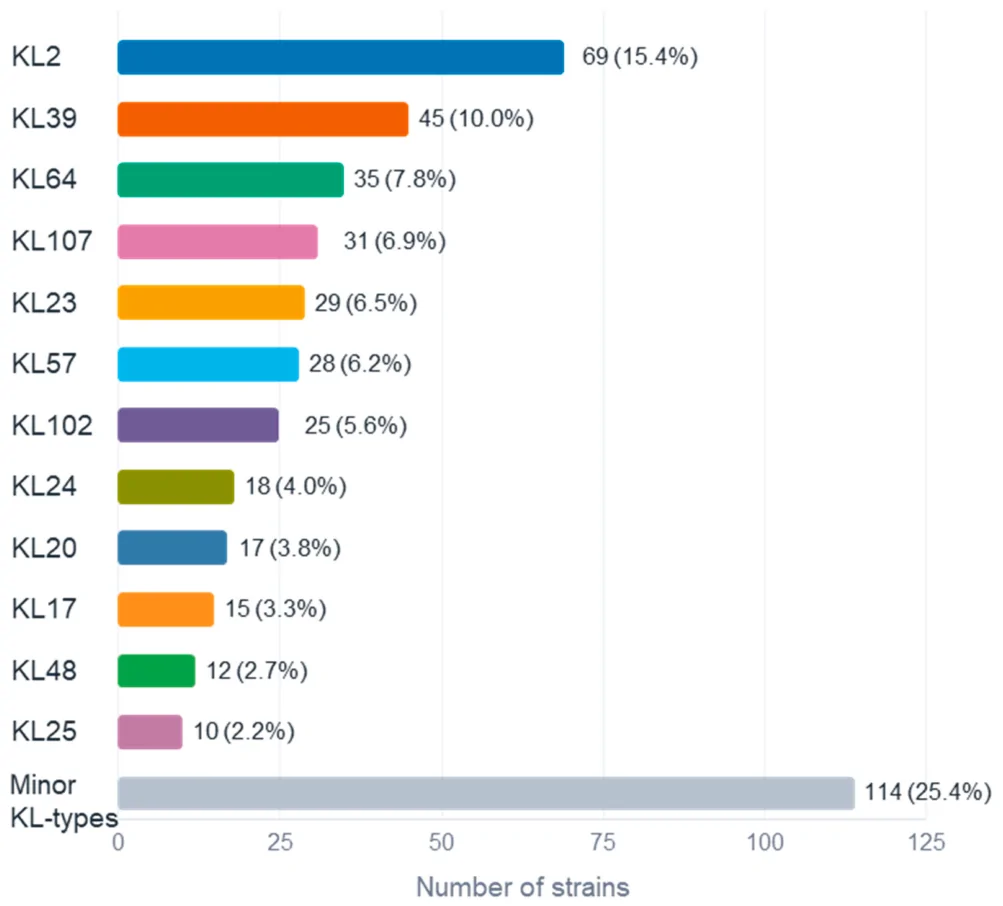
Capsule type distribution in the *K. pneumoniae* isolate collection. The minor KL types category included KL types represented by fewer than ten isolates.

Capsular typing based on *wzi* gene sequencing revealed 56 capsule locus (KL) types among the 448 isolates. Despite this diversity, 12 dominant KL types accounted for 74.5% of the collection, whereas each of the remaining 44 types represented less than 2% (Figure 1; Supplementary Table S1). The distribution of KL types showed no pronounced spatiotemporal bias. Contingency-table analysis identified statistically significant but weak associations with both medical centre (*χ*^2^ = 337.7, *df* = 132, *p* < 0.0001; Cramér’s V = 0.26) and year of isolation (*χ*^2^ = 223.0, *df* = 84, *p* < 0.0001; Cramér’s V = 0.27) (Supplementary Table S2).

### 2.2. Activity of Commercial Phage Cocktails Against the Complete K. pneumoniae Collection

**Figure 2:**
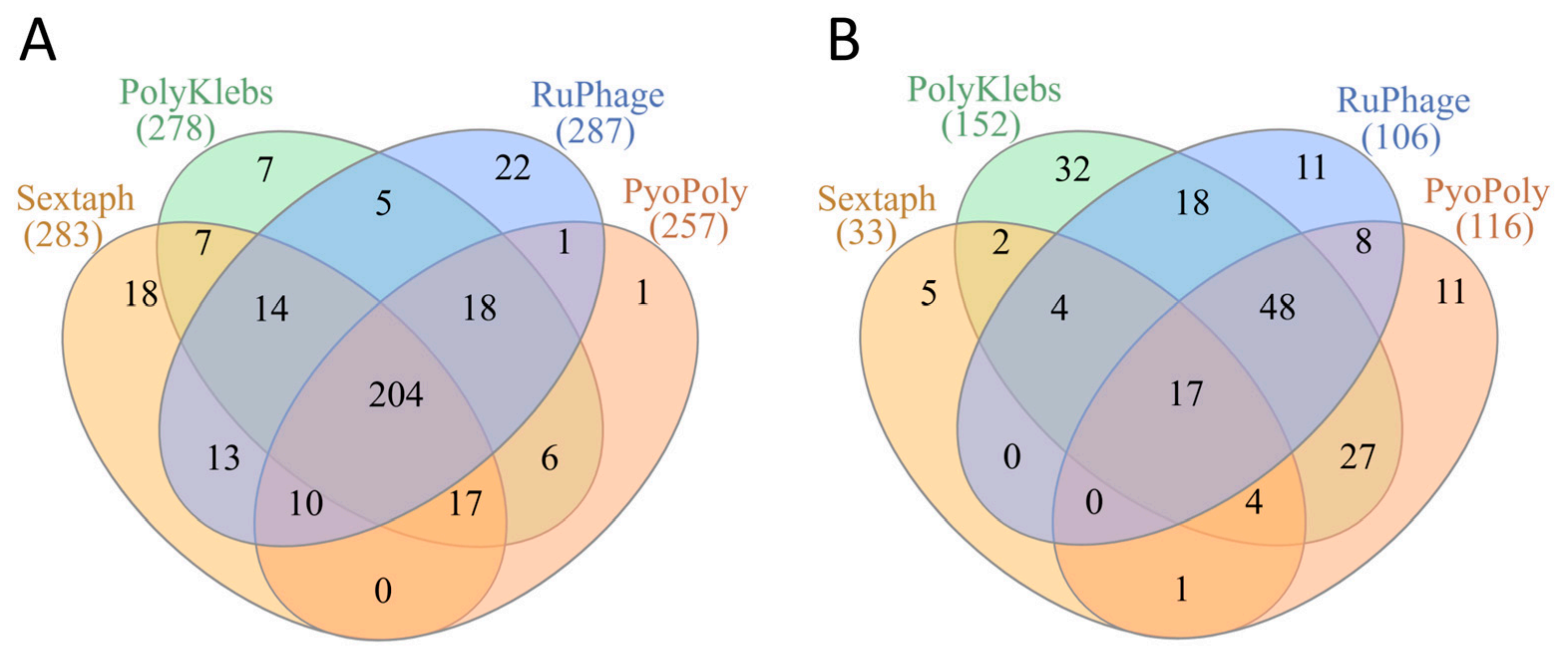
The activity of the bacteriophage cocktails according to host range (HR) (**A**) and putative therapeutic applicability (PTA) (**B**). Cocktail designations are as follows: Sextaph—Sextaphage; PolyKlebs—Purified Polyvalent *Klebsiella* Bacteriophage; RuPhage—Pyophage; and PyoPoly—Purified Polyvalent Pyobacteriophage.

The lytic activities of the commercial phage cocktails, assessed according to host range (HR; groups L, I and S), were broadly comparable. Individual cocktails lysed 57.4–64.1% of the isolates, and 86% of susceptible isolates were lysed by at least two cocktails (Figure 2A; Supplementary Table S1). However, differences among the preparations were more pronounced when activity was assessed according to putative therapeutic applicability (PTA; groups I and S; titre ≥10^6^ PFU/mL). Sextaph showed activity against 7.4% of the isolates, whereas the corresponding values for the other three cocktails ranged from 23.7% to 33.9%. All pairwise comparisons with Sextaph were statistically significant (*p* < 0.05) (Figure 2B; Supplementary Table S2).

Collectively, the four cocktails lysed 343 isolates according to the HR criterion. When the more stringent PTA criterion was applied, this number decreased to 187 isolates. The median difference between HR and PTA across the four cocktails was 35.9 percentage points (interquartile range (IQR), 30.6–44.3). The largest difference was observed for Sextaph, at 55.8 percentage points, whereas the differences for PolyKlebs, RuPhage and PyoPoly ranged from 28.1 to 40.4 percentage points.

To determine whether isolate origin was associated with cocktail activity, we compared the distribution of ordinal activity scores (R/L/I/S) among groups defined by medical centre and year of isolation. Analysis by medical centre identified statistically significant differences for Sextaph, PolyKlebs and PyoPoly using the Kruskal–Wallis test. Differences for RuPhage showed a trend towards significance (*p* = 0.0643) (Supplementary Table S2). In pairwise comparisons, isolates from the Federal Research and Clinical Center of Intensive Care Medicine and Rehabilitology and the Sklifosovsky Institute for Emergency Medicine were among those that most frequently differed significantly from isolates collected at other centres. Both HR and PTA were lower for isolates from these two institutions than for those from the other centres. Similar patterns were observed in the analysis by year of isolation. The distributions of lytic activity differed among years for all four cocktails, with isolates collected in 2019 being more susceptible to phages than those collected between 2021 and 2024.

Analysis of binary endpoints showed that HR (L + I + S versus R) was significantly associated with both medical centre and year of isolation for all four cocktails, whereas PTA (I + S versus R + L) was associated with these factors only for PyoPoly. The proportion of highly efficient lysis among HR-positive isolates (I + S versus L) differed significantly among medical centres and years for RuPhage and PyoPoly. For PolyKlebs, this association showed a trend towards significance (*p* = 0.0963).

### 2.3. Dependence of Commercial Cocktail Activity on K. pneumoniae Capsule Type

**Figure 3:**
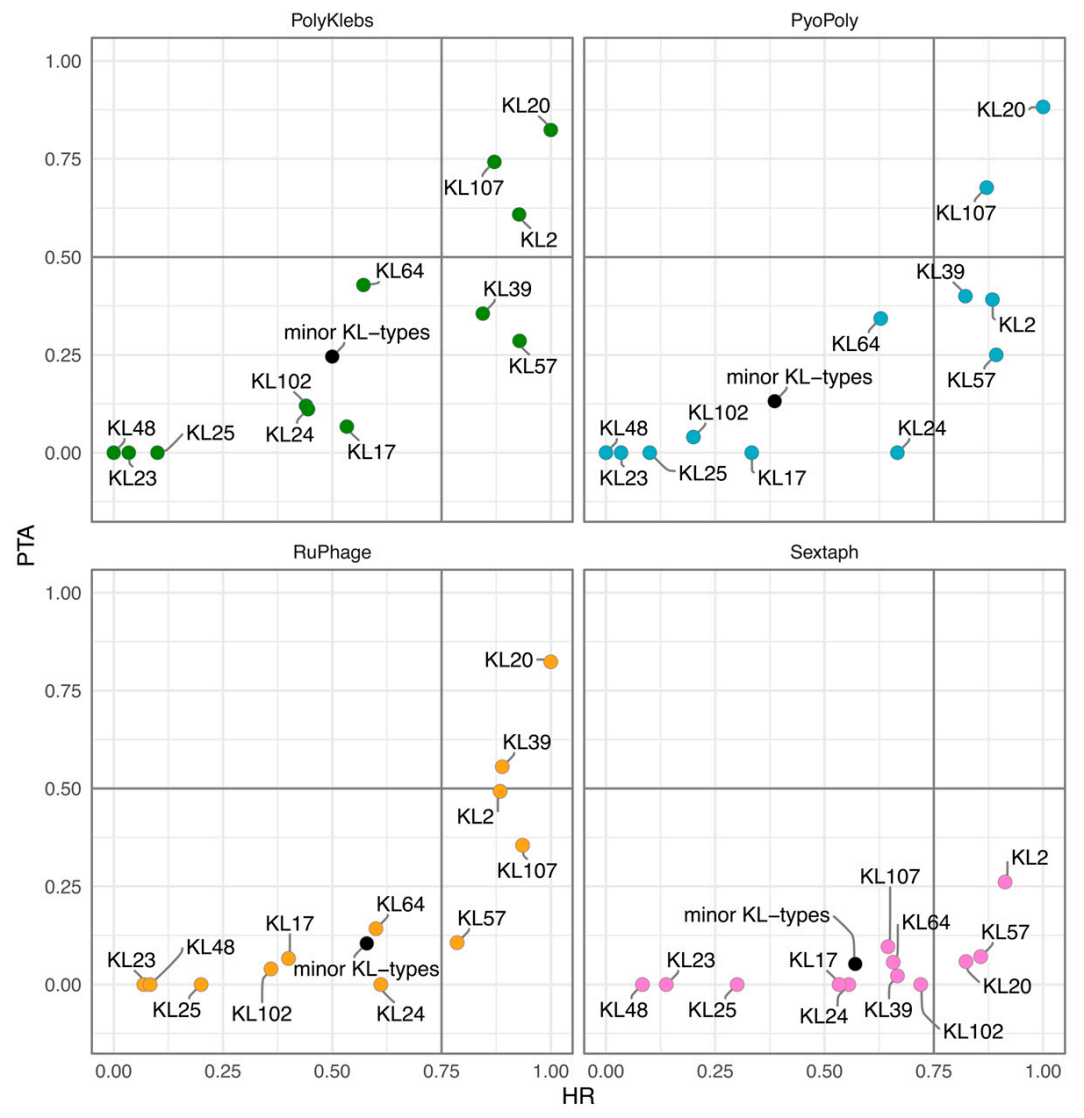
Activity of the phage cocktails against different *K. pneumoniae* capsule types according to host range (HR) and putative therapeutic applicability (PTA). Cocktail designations are as follows: Sextaph—Sextaphage; PolyKlebs—Purified Polyvalent *Klebsiella* Bacteriophage; RuPhage—Pyophage; and PyoPoly—Purified Polyvalent Pyobacteriophage.

To assess the relationship between cocktail activity and capsule type, we analysed the 12 dominant KL types represented by at least ten isolates (n ≥ 10), together with the combined minor KL-types group (Figure 3; Supplementary Table S3). For this analysis, HR ≥ 75% and PTA ≥ 50% were used as thresholds for good coverage and high putative therapeutic activity, respectively. Analysis of individual cocktail and KL-type combinations revealed substantial heterogeneity.

Both predefined thresholds were exceeded in only a limited number of cocktail–KL-type combinations. Most involved KL20, against which PolyKlebs, RuPhage and PyoPoly each achieved an HR of 100% and PTA values of 82–88%. Other combinations meeting both thresholds included PolyKlebs and PyoPoly against KL107, RuPhage against KL39 and PolyKlebs against KL2. The difference between HR and PTA was relatively small across these combinations, with a median of 20.8 percentage points (IQR, 17.6–29.5). However, HR and PTA did not coincide in any combination.

A more common pattern was an HR above 75% accompanied by a PTA below 50%. KL57 provided the clearest example of this pattern. All four cocktails showed high HR values against KL57 isolates, ranging from 79% to 93%, whereas PTA did not exceed 29%. The same pattern was observed for Sextaph, RuPhage and PyoPoly against KL2; PolyKlebs and PyoPoly against KL39; RuPhage against KL107; and Sextaph against KL20. Across these combinations, the median difference between HR and PTA was 64.3 percentage points (IQR, 49.1–66.5), indicating a substantial discrepancy between nominal coverage and highly efficient activity.

None of the cocktails achieved an HR of ≥75% against KL64, KL23, KL102, KL24, KL17, KL48, KL25 or the minor KL-types group. For KL23, KL48 and KL25, PTA was zero for all four cocktails. PTA was also frequently zero for KL24 and KL17, whereas PTA values remained low for KL102 and the minor KL-types group regardless of the cocktail tested. For these capsule types, activity was limited not only by the loss of highly efficient activity among isolates exhibiting lysis but also by insufficient nominal coverage of the corresponding KL groups.

Differences among the cocktails further indicated that capsule type alone did not fully determine the activity profile. Sextaph did not reach the PTA threshold of ≥50% for any KL type included in the analysis and was significantly less effective than the other cocktails against KL2, KL20, KL39 and KL107. Against KL102, the activity of Sextaph differed significantly from that of PyoPoly. Within the minor KL-types group, PolyKlebs was significantly more effective than PyoPoly (Supplementary Table S2).

Multivariable logistic regression showed that KL type remained the most reproducible factor associated with HR and PTA after adjustment for year of isolation and medical centre. KL2 was positively associated with susceptibility in all eight models, KL107 in seven models and KL39 in six models. KL64 was associated only with PTA for PolyKlebs and PyoPoly. By contrast, only individual contrasts involving year of isolation or medical centre were significant, and these associations did not form a consistent overall pattern.

### 2.4. Activity of Monophages and Commercial Cocktails Against KL2 K. pneumoniae Isolates

**Figure 4:**
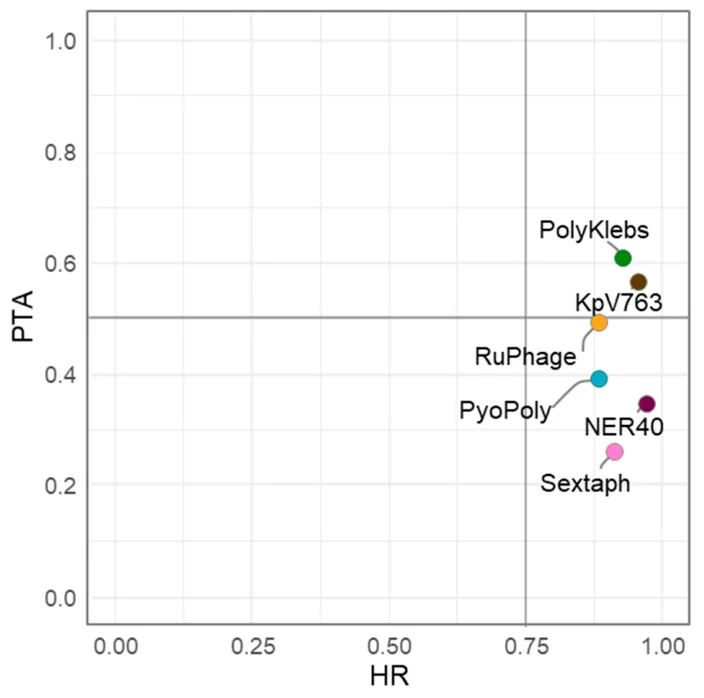
Activity of the phage cocktails and monophages against *K. pneumoniae* isolates with capsule type KL2, according to host range (HR) and putative therapeutic applicability (PTA). Cocktail designations are as follows: Sextaph—Sextaphage; PolyKlebs—Purified Polyvalent *Klebsiella* Bacteriophage; RuPhage—Pyophage; and PyoPoly—Purified Polyvalent Pyobacteriophage. NER40 and KpV763 are capsule-specific monophages.

A subset of 69 *K. pneumoniae* isolates with capsule type KL2 was used to compare the activities of commercial cocktails and capsule-specific monophages (Figure 4; Supplementary Table S3). The HR values of monophages NER40 and KpV763 were 95.7% and 97.1%, respectively. Among susceptible isolates, 91.3% were lysed by at least one monophage and at least one commercial cocktail. As observed for the commercial cocktails, PTA was lower than HR, reaching 34.8% for NER40 and 56.5% for KpV763.

KpV763 and PolyKlebs reached both predefined thresholds of HR ≥ 75% and PTA ≥ 50%. By contrast, NER40 and the remaining commercial cocktails provided good coverage but low putative therapeutic activity, with HR ≥ 75% and PTA < 50%. The differences between HR and PTA were 62.3 percentage points for NER40 and 39.1 percentage points for KpV763. These values were within the range observed for the commercial cocktails against KL2 isolates, which was 31.9–65.2 percentage points. Among the 69 KL2 isolates, 55 (79.7%) were HR-positive for all six preparations, whereas only four (5.8%) met the PTA criterion for all six.

In pairwise comparisons, both monophages, as well as PolyKlebs, RuPhage and PyoPoly, showed significantly greater putative therapeutic activity than Sextaph (*p* < 0.05). No statistically significant difference was detected between the two monophages or between either monophage and PolyKlebs (Supplementary Table S2).

In contrast to the analysis of the complete collection, the Kruskal–Wallis test identified no statistically significant differences in the distribution of ordinal lytic-activity scores (R/L/I/S) among medical centres or years of isolation for any cocktail or monophage tested. Analysis of the binary endpoints similarly showed no significant association of medical centre or year of isolation with HR, PTA or the proportion of highly efficient lysis among isolates exhibiting specific lysis.

## 3. Discussion

In this study, we addressed three related questions relevant to both the rational design and practical application of phage cocktails. First, how reliably does specific lysis observed in a spot test predict lytic activity at therapeutically relevant phage titres? Second, can the observed activity profiles be explained by limited receptor coverage alone? Third, to what extent can results obtained using a single local strain panel be extrapolated to other clinical populations?

As a methodological framework, we used two endpoints derived from semiquantitative Gratia titration. Host range (HR) comprised isolates against which a cocktail or monophage produced specific lysis, irrespective of the preparation dilution, and therefore reflected a detectable phage–bacterium interaction. Putative therapeutic applicability (PTA) comprised the subset of isolates against which lysis persisted at dilutions corresponding to a titre of ≥10^6^ PFU/mL in the original preparation. This threshold was chosen as a pragmatic operational criterion, reflecting the lower end of phage concentrations used in clinical studies, including treatments with favorable outcomes [5,23,24,25,26]. Although many studies employ substantially higher concentrations [27,28,29,30], therapeutic doses vary according to the infection site, route of administration and treatment protocol, and no universal *in vitro* standard exists. The 10^6^ benchmark therefore serves as a reproducible endpoint for comparing phage preparations within this study, not as a clinical breakpoint or predictor of therapeutic efficacy.

These endpoints were evaluated using a clinically relevant collection of *K. pneumoniae* isolates obtained from several medical centres over an eight-year period and stratified by capsule type. The collection included both globally prevalent and regionally important KL types [31,32,33]. Moreover, the distribution of capsule types showed no pronounced spatiotemporal bias.

At the level of the complete collection, all four commercial cocktails exhibited similarly broad HR values. However, stratification by capsule type revealed substantial heterogeneity in coverage. For several epidemiologically important KL types, including KL2, KL20, KL39, KL57 and KL107, HR exceeded 75%, and the median proportion of isolates without detectable specific lysis was only 11.6% (interquartile range (IQR), 7.2–16.1). For less extensively covered KL types, including KL23, KL64 and KL102, this proportion reached 45.6% (IQR, 39.7–57.0). Some capsule types, such as KL48, were not lysed by any of the cocktails tested (Supplementary Table S3).

Incomplete coverage of capsule types against which the preparations contain active phages may have several explanations. The exposure and chemical modification of capsular polysaccharides, including acetylation, can vary among strains of the same KL type, rendering the receptor inaccessible to some phage depolymerases [34,35]. Mutations or changes in capsule biosynthesis may also prevent adsorption or confer cross-resistance to phages using the same receptor. In addition to capsule-related changes, alterations in other surface structures, such as high-molecular-weight lipopolysaccharide (LPS), may impair phage adsorption or affect subsequent steps of infection [36,37,38]. Furthermore, phage depolymerases themselves can differ in substrate specificity, contributing to variability in host range even among strains sharing the same capsule type [39]. Finally, in some isolates, intracellular antiphage defence systems may completely block phage replication even after successful adsorption; however, at the level of HR, this phenotype cannot be distinguished from a failure of adsorption.

Application of the more rigorous PTA criterion revealed a further reduction in cocktail activity, consistent with previous studies [7,10,40]. This analysis demonstrated that HR systematically overestimated the proportion of isolates against which high lytic activity was achieved. The magnitude of the difference depended on both capsule type and phage preparation, ranging from 11.8 percentage points for PyoPoly against KL20 to 78.6 percentage points for Sextaph against KL57. The lytic spectra of the individual cocktails also diverged substantially at the level of PTA despite being similar at the level of HR. Cocktails with similar nominal host ranges are therefore not necessarily functionally interchangeable, and selecting a cocktail solely on the basis of specific lysis could result in the prescription of a less active preparation.

Comparison of the commercial cocktails with capsule-specific monophages against KL2 isolates showed that the HR–PTA difference persisted even when monophages were used at a high initial titre (10^9^ PFU/mL). Importantly, all 69 KL2 isolates were HR-positive for at least one preparation, and 55 were HR-positive for all six, whereas only four met the PTA criterion for all tested preparations, highlighting a marked discordance between detectable lysis and high-titre activity. Nominal capsule-type matching therefore did not ensure high-titre activity, and the persistence of the HR–PTA difference under these conditions indicates that it cannot be explained solely by unknown cocktail composition, competition among phages or insufficient component titres.

Collectively, our findings support a funnel model in which the lytic spectrum of a cocktail progressively narrows from the complete strain panel to nominal coverage defined by HR and subsequently to highly efficient activity defined by PTA. At the first stage, isolates that do not exhibit specific lysis are excluded from the activity spectrum. The proportion of such isolates varied from 11% to 45%, depending on the capsule type. This phenotype may reflect the absence or inaccessibility of an appropriate receptor or the complete suppression of infection after adsorption. At the second stage, some HR-positive isolates fail to meet the PTA criterion, producing an additional functional loss of 28 to 56 percentage points. Since these isolates already exhibited specific lysis, receptor incompatibility alone cannot account for this loss. Moreover, the persistence of this loss with high-titre monophages, combined with the divergence of PTA profiles despite highly concordant HR, suggests that post-adsorption limitations contribute to the overall pattern. However, this remains a functional inference rather than a mechanistic conclusion: reduced adsorption efficiency and insufficient titres of individual active components within the commercial cocktails cannot be excluded, and intracellular antiphage mechanisms were not assessed directly. HR therefore primarily characterizes the breadth of nominal coverage, whereas the transition from HR to PTA quantifies the depth of lytic activity.

This model also provides a framework for assessing the extent to which findings obtained using one local strain panel can be extrapolated to other clinical populations. KL type was the most consistent predictor of activity after adjustment for medical centre and year of isolation. As capsule type defines the principal adsorption target for many *K. pneumoniae* phages, the capsule-type distribution of the target population should therefore be a primary consideration when extrapolating cocktail activity between clinical collections. The additional functional loss between HR and PTA is less readily extrapolated. When receptor variation was restricted to the KL2 subset, neither cocktail nor monophage activity showed detectable associations with spatiotemporal characteristics. This pattern is consistent with the contributing factors being heterogeneously distributed and acting at the level of specific phage–strain combinations, potentially including post-adsorption limitations. However, the absence of significant associations may also reflect the smaller size of the KL2 subset. In either case, extrapolation to other capsule types requires caution.

Our findings have two practical implications. First, lysis produced by an undiluted preparation is insufficient to assess its potential activity. Quantitative Gratia titration against the isolate under investigation is preferable. If testing a complete dilution series is not feasible, the minimum requirement should be to test the dilution corresponding to a predefined threshold of *in vitro* activity (≥10^6^ PFU/mL in this study). In this study, that threshold was equivalent to ≥10^6^ PFU/mL in the original preparation. Second, rational cocktail design should primarily account for receptor matching, as emphasized in previous studies [8,9,41,42], but should also consider additional factors that may reduce activity, including the ability of phages to overcome antiphage defence systems [15,17] and the potential for competition among phages [16,43]. Although approaches for analysing these factors are beginning to emerge for individual pathogens, substantial gaps remain in our understanding of the fundamental mechanisms governing phage–host interactions in many bacterial species.

This study has several limitations. First, the manufacturers do not disclose the precise composition of the commercial cocktails or the titres of their individual active components. We therefore could not directly assess the contribution of the phage repertoire and/or titers to the observed reduction in activity. Second, we did not measure adsorption directly. Consequently, among HR-positive isolates that did not meet the PTA criterion, we were unable to distinguish between reduced adsorption efficiency and limitations acting at stages subsequent to adsorption. Third, bacterial isolates were characterized by *wzi* typing rather than whole-genome sequencing. We therefore could not assess antiphage defence systems or strain clonality, while capsule expression and receptor accessibility were not evaluated phenotypically.

## 4. Materials and Methods

### 4.1. Clinical Collection of Klebsiella pneumoniae Isolates

The study included a collection of 448 clinical *K. pneumoniae* isolates maintained at the Lopukhin Federal Research and Clinical Center of Physical-Chemical Medicine of the Federal Medical Biological Agency. The isolates had been collected from 12 medical institutions between 2018 and 2025 (Supplementary Table S1).

Bacterial strains were cultured in LB medium (Miller, Condalab, Madrid, Spain) at 37 °C. Species identity was confirmed using matrix-assisted laser desorption/ionization time-of-flight mass spectrometry (MALDI-TOF) [44]. Capsule typing was performed by sequencing the *wzi* gene [45].

### 4.2. Commercial Phage Cocktails and Monophages

Four commercial phage cocktails manufactured by NPO Microgen (Moscow, Russia) were used in this study: Sextaphage (Sekstafag; batch П267, manufactured in October 2022, Perm, Russia), Purified Polyvalent Klebsiella Bacteriophage (Bakteriofag klebsiell polivalentnyj ochishchennyj; batch У19, manufactured in May 2022, Ufa, Russia), Pyophage (Piofag; batch H0580123, manufactured in January 2023, Nizhny Novgorod, Russia), and Purified Polyvalent Pyobacteriophage (Piobakteriofag polivalentnyj ochishchennyj; batch У21, manufactured in July 2022, Ufa, Russia). Throughout the manuscript, these products are designated Sextaph, PolyKlebs, RuPhage, and PyoPoly, respectively. The lytic activity spectra stated in the manufacturers’ instructions are presented in Supplementary Table S1. The cocktails were stored at 6 °C in accordance with the manufacturer’s recommendations and were used within their stated shelf lives.

The monophages were obtained from the collection of the Lopukhin Federal Research and Clinical Center of Physical-Chemical Medicine of the Federal Medical Biological Agency. Bacteriophage NER40 (*Drulisvirus* genus; GenBank accession MZ602146.1) was isolated from the Chermyanka River in northern Moscow in June 2018 [46]. Bacteriophage KpV763 (*Przondovirus* genus; GenBank accession OR639934.1) was isolated from the sewage system of Clinical Hospital No. 123 in Odintsovo in April 2021. Bacteriophage titres were determined using the double-agar-layer method [47] and were 10^9^ PFU/mL. Purified monophage lysates were stored at 6 °C.

### 4.3. Assessment of Bacteriophage Activity

The activity of the phage cocktails and monophages was assessed using the Gratia titration method with minor modifications [47]. An aliquot of 100 µL of an overnight culture of each test strain was incorporated into a soft LB agar (0.7% agar) overlay. Aliquots of 5 µL of each cocktail or monophage dilution (10^0^, 10^−2^, 10^−4^ and 10^−6^) were then applied to the agar surface. Phage activity was evaluated by examining the resulting lysis zones after overnight incubation at 37 °C.

On the basis of their susceptibility to the cocktails and monophages, *K. pneumoniae* strains were assigned to four activity groups. Strains were classified as:

- R (resistant; score 0) when no lysis was observed or when only non-specific lysis was present, defined as a turbid zone without confluent lysis;
- L (low lysis efficacy; score 1) when lysis zones and/or individual plaques were observed at the 10^0^ and 10^−2^ dilutions;
- I (intermediate lysis efficacy; score 2) when lysis zones and/or individual plaques were observed at the 10^−4^ dilution;
- S (susceptible to phage; score 3) when lysis zones and/or individual plaques were observed at the 10^−6^ dilution.

All phage susceptibility assays were performed in three independent biological replicates. The final R/L/I/S classification was assigned based on the mean endpoint dilution across replicates, rounded to the nearest tested dilution.

Two complementary endpoints were used for the biological interpretation of cocktail and monophage activity. Host range (HR) was defined as the proportion of isolates exhibiting specific lysis and included isolates assigned to the L, I and S groups. Putative therapeutic applicability (PTA) was defined as the proportion of isolates against which high lytic activity was achieved and included isolates assigned to the I and S groups.

### 4.4. Statistical Analysis and Data Presentation

To assess potential spatiotemporal bias in the isolate collection, contingency tables were constructed for KL type by year of isolation and KL type by medical centre. To reduce artefacts arising from sparse data, KL types were grouped into 12 dominant categories, each representing ≥2% of the collection, and a combined minor KL-types category comprising the remaining 44 types. Associations were evaluated using Pearson’s *χ*^2^ test, and their strength was quantified using Cramér’s V.

The activity of the phage cocktails was compared using non-parametric analysis of variance for matched samples with the Friedman test. Pairwise comparisons between cocktails were performed on the basis of mean ranks, with the false discovery rate (FDR) controlled using the method of Benjamini, Krieger and Yekutieli.

The ordinal activity scale comprising the R, L, I and S categories, corresponding to scores of 0–3, was also analysed to determine whether the lytic activity of the phage cocktails and monophages was associated with medical centre or year of isolation. Score distributions were compared among independent groups using the Kruskal–Wallis test. Pairwise comparisons were performed with FDR control using the two-stage linear step-up procedure of Benjamini, Krieger and Yekutieli.

Associations of medical centre or year of isolation with HR, PTA and the proportion of highly efficient lysis among isolates exhibiting specific lysis were assessed using Pearson’s *χ*. Contingency tables were constructed for three binary endpoints: HR, defined as L + I + S versus R; PTA, defined as I + S versus R + L; and highly efficient lysis among HR-positive isolates, defined as I + S versus L.

For analyses of the complete collection using the Kruskal–Wallis and Pearson’s *χ*^2^ tests, isolates from medical centres that contributed fewer than 20 isolates were combined into an “Other” category to reduce group sparsity. In analyses stratified by year of isolation, isolates collected in 2018 (*n* = 12) and 2020 (*n* = 1) were excluded because of the small sample sizes.

The subset of isolates with capsule type KL2 was analysed using the same approach. For analyses stratified by medical centre, institutions that contributed ≥10 KL2 isolates were analysed separately, whereas the remaining institutions were combined into an “Other” category (*n* = 25). Analyses stratified by year included isolates collected in 2019 (*n* = 12), 2021 (*n* = 10), 2022 (*n* = 14), 2023 (*n* = 14) and 2024 (*n* = 17). Isolates collected in 2018 were excluded because only two KL2 isolates were available (*n* = 2).

Statistical tests were performed using GraphPad Prism 8.4.3 and OriginPro 2021.

Associations between medical centre, sampling year and KL type and the binary HR and PTA outcomes for Sextaph, PolyKlebs, RuPhage and PyoPoly were evaluated using eight separate multivariable logistic regression models. A value of 1 was modelled as the event. All three predictors were included simultaneously as categorical covariates, without interaction terms. Within each predictor, levels represented by fewer than 30 observations were pooled as “Other” to reduce sparse-data separation and model-matrix singularity. Treatment coding used the most frequent post-pooling level as the reference category: Gorbacheva for medical centre, 2024 for year and Other for KL type. All 448 observations had complete data and were included in every model.

Models were fitted by maximum likelihood using the Logit implementation in statsmodels (Python v. 3.9.7), with a maximum of 200 Newton–Raphson iterations. Regression coefficients and their 95% Wald confidence limits were exponentiated to obtain adjusted odds ratios and 95% confidence intervals. Two-sided Wald *z*-test *p*-values were reported without adjustment for multiple comparisons. Model fit was summarized using McFadden pseudo-R^2^ and the Akaike information criterion.

Venn diagrams were generated using InteractiVenn [48]. The distribution of capsule types within the isolate collection was visualized using Matplotlib v. 3.10.3 [49]. Host-range and putative therapeutic applicability profiles of the phage cocktails and monophages were visualized using ggplot2 v. 3.5.2 [50].

## 5. Conclusions

This study identified two sequential levels of restriction that account for the difference between detectable lysis and highly efficient activity of phage preparations. The first level corresponds to nominal coverage, as measured by HR, and encompasses the factors that prevent specific lysis, including the absence or inaccessibility of an appropriate receptor and suppression of infection after adsorption. The second level corresponds to the transition from HR to PTA, suggesting that post-adsorption limitations are a likely and potentially substantial contributor to this functional loss. This interpretation represents a mechanism-based hypothesis derived from the phenotypic data, but the contribution of post-adsorption limitations remains to be quantified using direct adsorption measurements and intracellular infection assays.

Because HR describes only the nominal lytic spectrum, the detection of specific lysis cannot reliably predict highly efficient activity or establish the underlying mechanism of resistance. A spot test should therefore not be used as the sole criterion for assessing potential therapeutic applicability. Serial-dilution titration against the isolate under investigation is the preferred approach. When only a reduced testing protocol is feasible, at least the dilution corresponding to a predefined threshold of *in vitro* activity should be tested.

The same principle applies to the rational design of phage cocktails. In addition to selecting phages according to their adsorption spectra, cocktail design should include functional characterization of each component at the level of productive infection, including its capacity to overcome intracellular barriers.

## Data Availability

The raw data supporting the conclusions of this article will be made available by the authors on request.

## Supplementary Materials

The following supporting information can be downloaded at: https://www.mdpi.com/article/10.3390/antibiotics15090900/s1. Supplementary Table S1. Metadata, molecular typing, and phage lysis profiles of the *K. pneumoniae* clinical isolate collection; Supplementary Table S2. Detailed results of omnibus and post hoc analyses of phage activity in the complete *Klebsiella pneumoniae* collection and the KL subset; Supplementary Table S3. Host range and putative therapeutic applicability of commercial phage cocktails stratified by *Klebsiella pneumoniae* capsule type.
